# Patellar Osteomyelitis Mimicking Cellulitis Successfully Treated Without Surgical Intervention

**DOI:** 10.7759/cureus.117828

**Published:** 2026-10-12

**Authors:** Aki Nakamura, Akihiro Iguchi, Katsuhiko Kitazawa

**Affiliations:** 1 Department of Pediatrics, University of Tokyo, Tokyo, JPN; 2 Department of Pediatrics, Asahi General Hospital, Asahi, JPN

**Keywords:** bacteremia, cellulitis, magnetic resonance imaging, patella, pediatric osteomyelitis

## Abstract

Osteomyelitis is an inflammatory infection of the bone whose incidence in children has increased over the past decade. It often presents with nonspecific clinical manifestations, and it is often difficult to diagnose. Here, we report the case of a school-aged boy with patellar osteomyelitis caused by methicillin-susceptible *Staphylococcus aureus* (MSSA) complicated by bacteremia. Blood cultures obtained on admission grew Gram-positive cocci, later identified as MSSA. Magnetic resonance imaging (MRI) revealed areas of high intensity in the left patella and adjacent overlying soft tissues on short tau inversion recovery sequences. Despite his relatively mild clinical presentation, prompt magnetic resonance imaging and blood cultures enabled an early and accurate diagnosis, allowing successful antimicrobial treatment without surgical intervention. This case highlights that in children presenting with unilateral knee pain, patellar osteomyelitis should be considered in the differential diagnosis, and early MRI may facilitate prompt diagnosis and help avoid invasive surgery.

## Introduction

Osteomyelitis is an infectious, inflammatory condition affecting bone tissue. In northern European countries, such as Germany, its pediatric incidence rose by approximately 10% between 2009 and 2019 [1], a trend that may partly reflect advances in microbiological diagnosis and improved access to diagnostic tools. Delayed diagnosis can lead to significant long-term complications; thus, timely identification is essential.

Acute osteomyelitis typically involves the metaphysis of long bones, particularly the femur and tibia. By contrast, sesamoid bones such as the patella are rarely affected, accounting for less than 1% of pediatric cases [2]. Differentiating patellar osteomyelitis from more common conditions, such as cellulitis or septic arthritis, is particularly challenging due to overlapping symptoms and the cartilage-predominant structure of the pediatric patella [3], which delays early radiographic detection. Consequently, diagnostic delays often lead to disease progression [4].

Current clinical guidelines for pediatric osteomyelitis recommend conservative medical management with targeted antimicrobial therapy as the primary treatment, reserving surgical intervention for cases with abscess formation or severe disease progression [5].

In this report, we present the case of a school-aged male patient with patellar osteomyelitis caused by methicillin-susceptible* Staphylococcus aureus *(MSSA), complicated by bacteremia. Despite the diagnostic difficulty due to the relatively mild nature of his clinical manifestations, early magnetic resonance imaging (MRI) in conjunction with blood culture allowed prompt recognition and successful conservative management with antimicrobial therapy alone.

## Case presentation

A previously healthy eight-year-old boy was admitted to the hospital because of fever and difficulty walking following an 11-day history of left knee pain. Three weeks before admission, he had sustained a minor contusion to the knee during a fall. On admission, the patient was able to ambulate independently, presenting with only a mild antalgic gait due to left knee pain. He presented with a mild fever, but no other significant abnormalities in his vital signs were observed, as summarized in Table 1.

**Table 1:** Vital signs on admission

Vital sign	Value
Body temperature	37.9 ℃
Systolic blood pressure	104 mmHg
Heart rate	121 beats/min
Respiratory rate	24 breaths/min

Physical examination revealed focal tenderness and swelling over the anterior aspect of the left knee without erythema. The range of motion of the knee was limited, but the patellar ballottement test was negative. Laboratory examinations showed mildly elevated inflammatory markers, including white blood cell count and C-reactive protein, while immunoglobulin levels remained within the normal range, as detailed in Table 2.

**Table 2:** Laboratory examinations on admission WBC, white blood cell; Hb, Hemoglobin; Plt, Platelet count; CRP, C-reactive protein; TP, total protein; Alb, albumin; AST, aspartate aminotransferase; ALT, alanine aminotransferase; BUN, blood urea nitrogen; Cre, creatinine; IgG, Immunoglobulin G; IgA, Immunoglobulin A; IgM, Immunoglobulin M

Laboratory examinations	Reference range	Value on admission
WBC (×10^3^ /μL)	3.3−8.6	11.4
Neutrophils (%)	45.2−68.8	59.9
Lymphocytes (%)	26.8−43.8	27.0
Monocyte(%)	2.7−7.9	8.8
Eosinocyte(%)	0−10.0	3.6
Basophils(%)	0−5.0	0.7
Hb (g/dL)	13.7−16.8	13.5
Plt (×10^4^ /μL)	15.8−34.8	30.5
CRP (mg/dL)	＜ 0.14	0.85
TP(g/dL)	6.6−8.1	7.1
Alb(g/dL)	4.1−5.1	4.3
AST(IU/L)	13−30	21
ALT(IU/L)	10−42	8
BUN(IU/L)	8−20	13
Cre(mg/dL)	0.27−0.53	0.41
IgG (mg/dL)	861−1,747	890
IgA (mg/dL)	93−393	155
IgM (mg/dL)	33−183	145

Plain radiographs of the left knee were unremarkable. Ultrasonography performed by an orthopedic surgeon revealed no joint effusion or bursal effusion. Knee aspiration yielded no synovial fluid. Based on these findings, prepatellar cellulitis was suspected, and intravenous cefazolin (CEZ) at 90 mg/kg/day was initiated.

On the following day, the patient became afebrile, and his knee pain had improved. On hospital day 3, a single aerobic blood culture bottle was drawn on admission and grew Gram-positive cocci, later identified as MSSA. Antimicrobial susceptibility testing confirmed that the isolated MSSA strain was susceptible to CEZ. Following the identification of MSSA bacteremia on hospital day 3, echocardiography was performed, which revealed no valvular vegetations or signs of infective endocarditis. MRI revealed areas of high signal intensity in the left patella and adjacent overlying soft tissues on short tau inversion recovery sequences (Figure 1a, 1b), findings consistent with patellar osteomyelitis accompanied by knee cellulitis.

**Figure 1:**
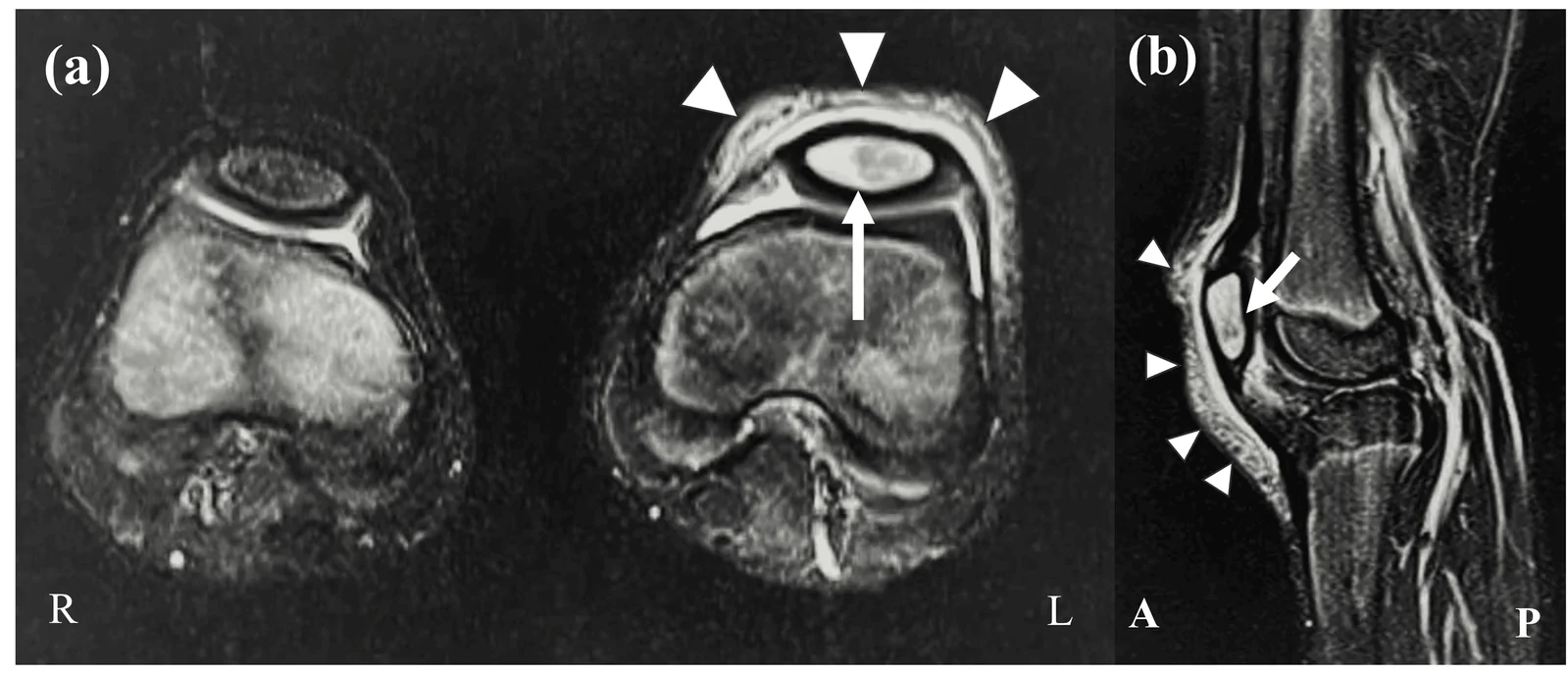
Magnetic resonance imaging (MRI) obtained on hospital day 3 after initiation of treatment (a) Axial and (b) sagittal MRI obtained on day 3 after initiation of treatment demonstrating high signal intensity on short tau inversion recovery (STIR) sequences at the left patella (arrow) and the adjacent overlying soft tissues (arrowheads). No evidence of subperiosteal or intraosseous abscess formation, nor was any sequestrum identified. No obvious abnormalities were observed in the right knee joint or the patella.

The CEZ dose was increased to 150 mg/kg/day as definitive antimicrobial therapy for MSSA bacteremia. Follow-up blood culture (a single aerobic blood culture bottle) performed on hospital day 3 confirmed a negative result. Over the subsequent nine days, tenderness and swelling of the left knee steadily decreased. WBC and CRP levels were immediately normalized after initiating antimicrobial treatment, as shown in Figure 2.

**Figure 2:**
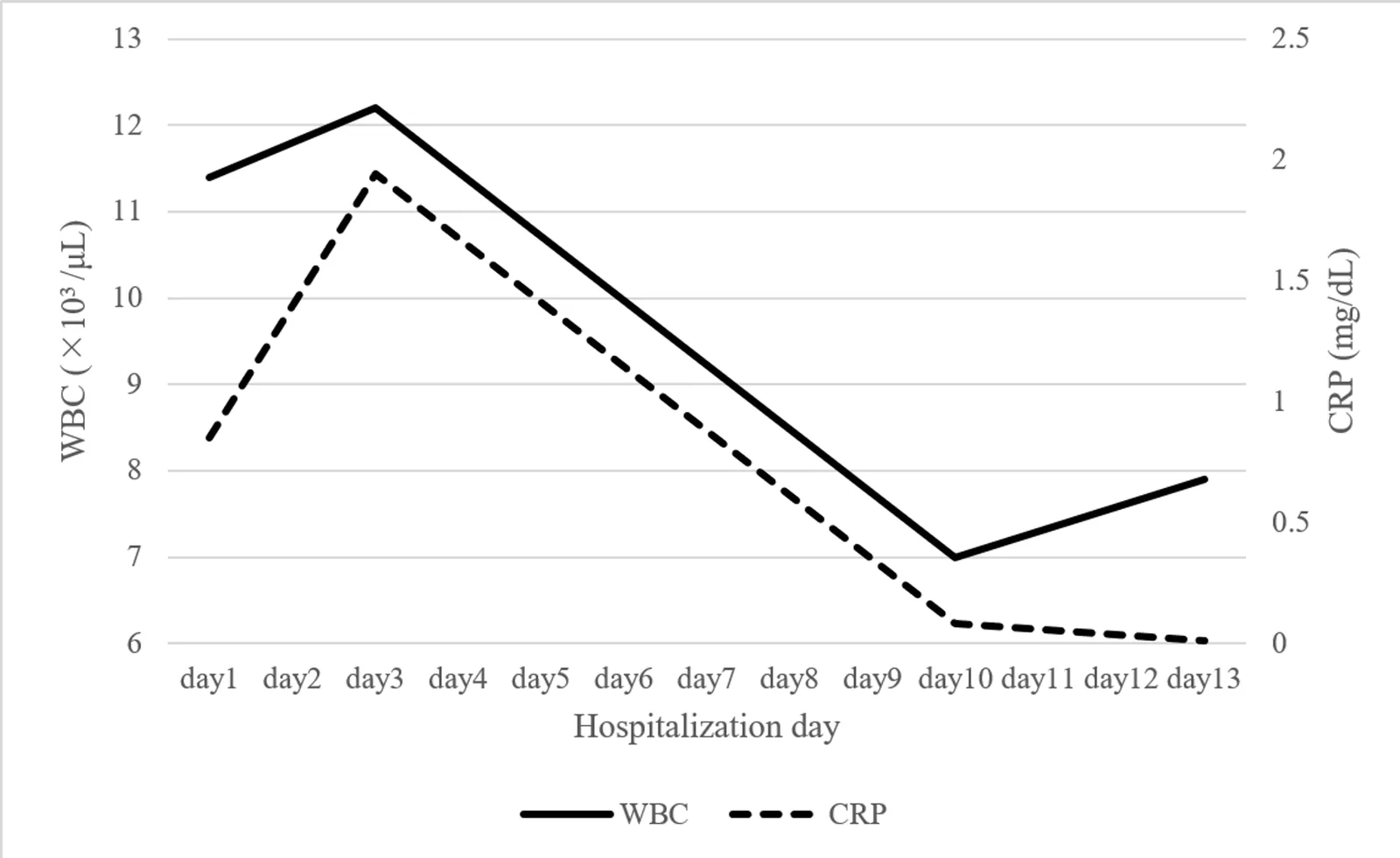
White blood cell (WBC) and C-reactive protein (CRP) values during hospitalization Figure 2 revealed that both inflammatory markers were immediately decreased after initiating antimicrobial treatment.

However, follow-up plain radiographs obtained on hospital day 15 demonstrated osteolytic changes in the left patella (Figure 3). The orthopedic team was re-consulted for further evaluation and management.

**Figure 3:**
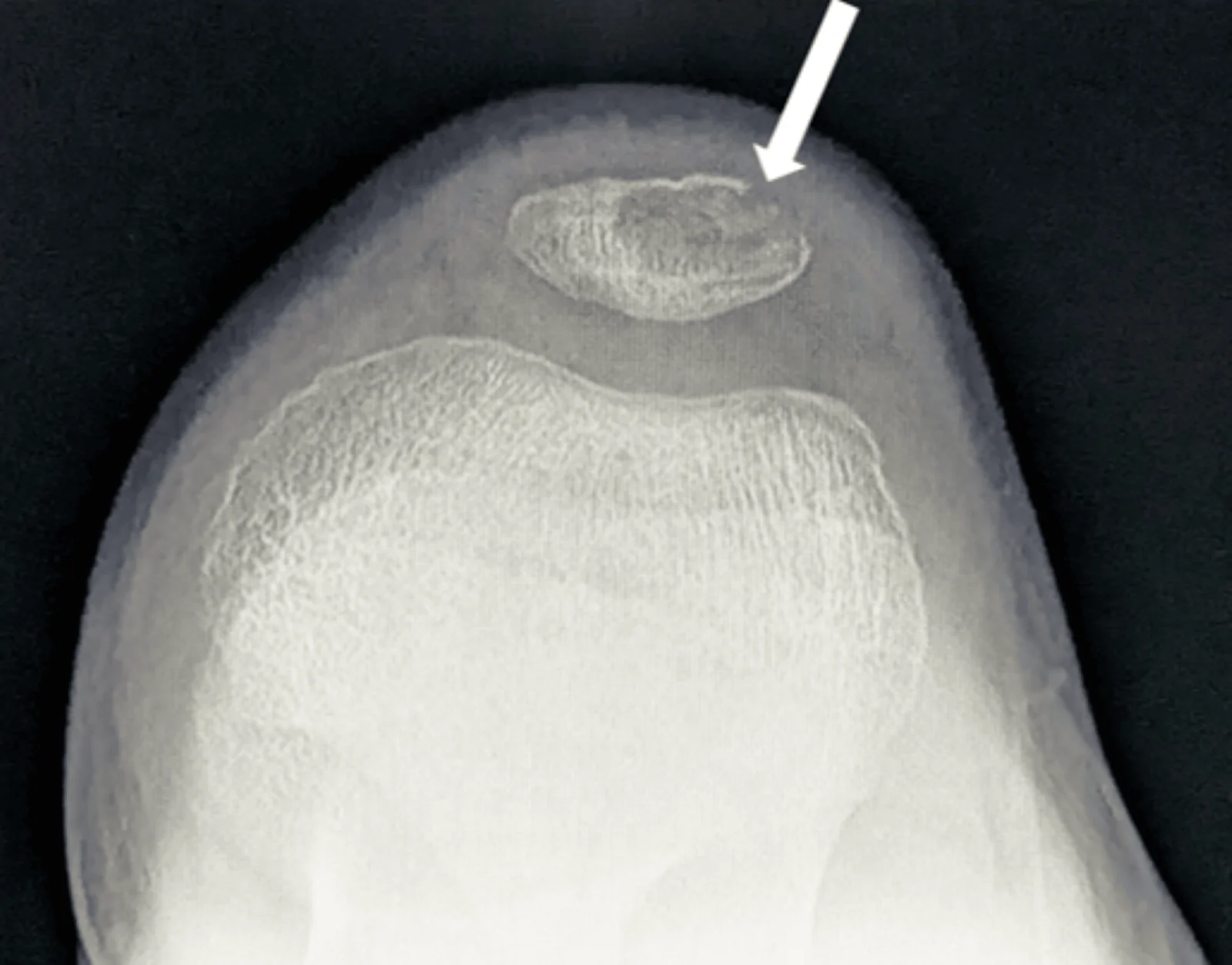
Plain radiography obtained on hospital day 15 after initiation of treatment demonstrating an osteolytic lesion in the left patella (arrow).

On hospital day 17, computed tomography (CT) showed cortical disruption of the anterior left patella with an associated cancellous bone defect (Figure 4).

**Figure 4:**
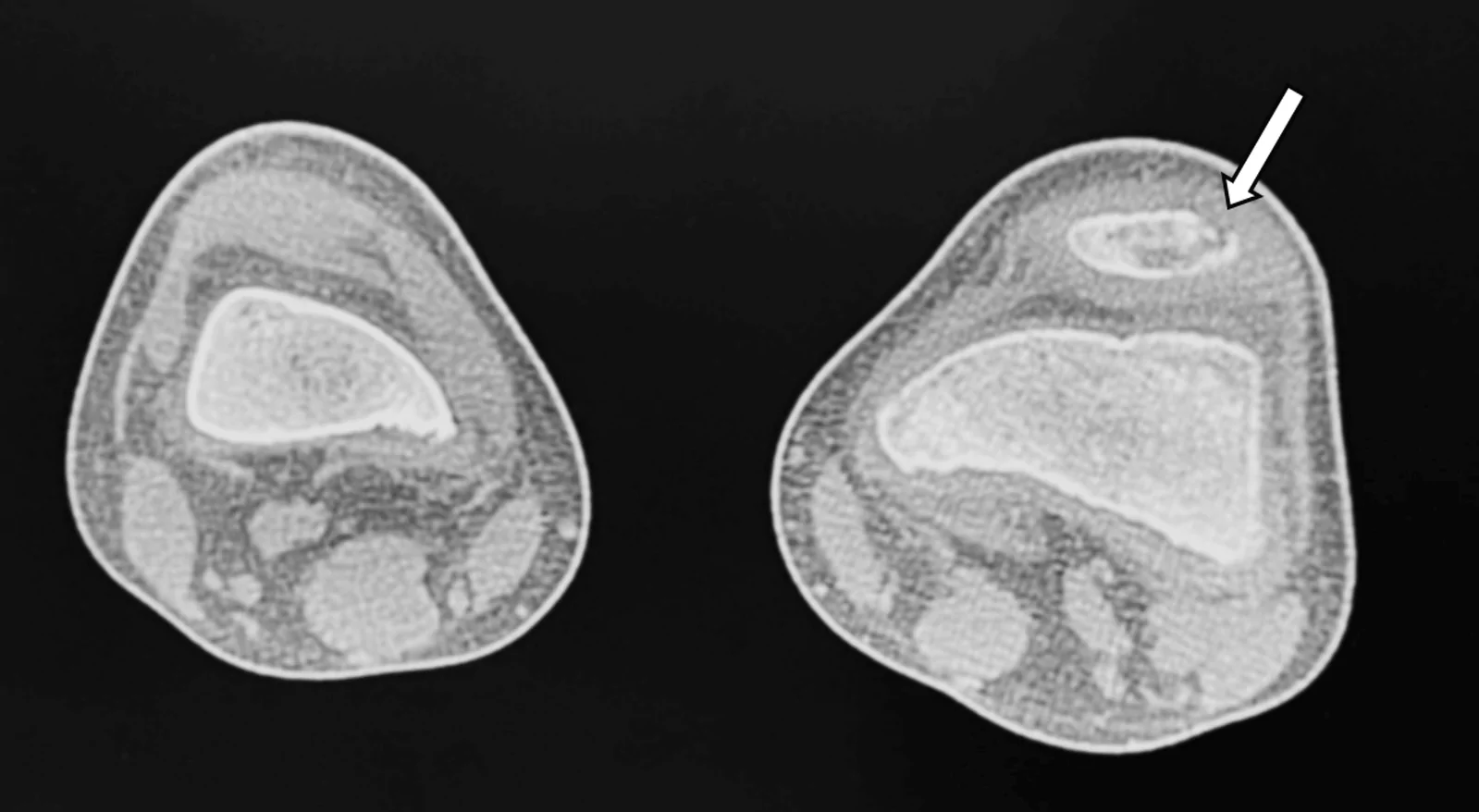
Computed tomography (CT) performed on hospital day 17 after initiation of treatment Figure 4 revealed cortical disruption of the anterior aspect of the left patella with an associated cancellous bone defect (arrow).

Although these findings were compatible with patellar osteomyelitis, in light of the favorable clinical course and normalization of CRP levels, the orthopedic team recommended continuation of conservative medical management while surgical curettage was being considered.

After excluding other infectious sources, including endocarditis, CEZ was switched to oral cephalexin at 50 mg/kg/day divided into three doses as effective step-down therapy, and the patient was discharged on hospital day 18. Intravenous antibiotic therapy was administered for 16 days, followed by oral antibiotics for a total treatment duration of 6 weeks. Because follow-up blood cultures showed no growth and the CRP level normalized, alongside marked clinical improvement, conservative medical treatment was continued. Plain radiographs obtained at the end of treatment demonstrated resolution of the osteolytic defect in the left patella (Figure 5). At 10 months after completion of therapy, the patient remained symptom-free with full range of motion of the left knee.

**Figure 5:**
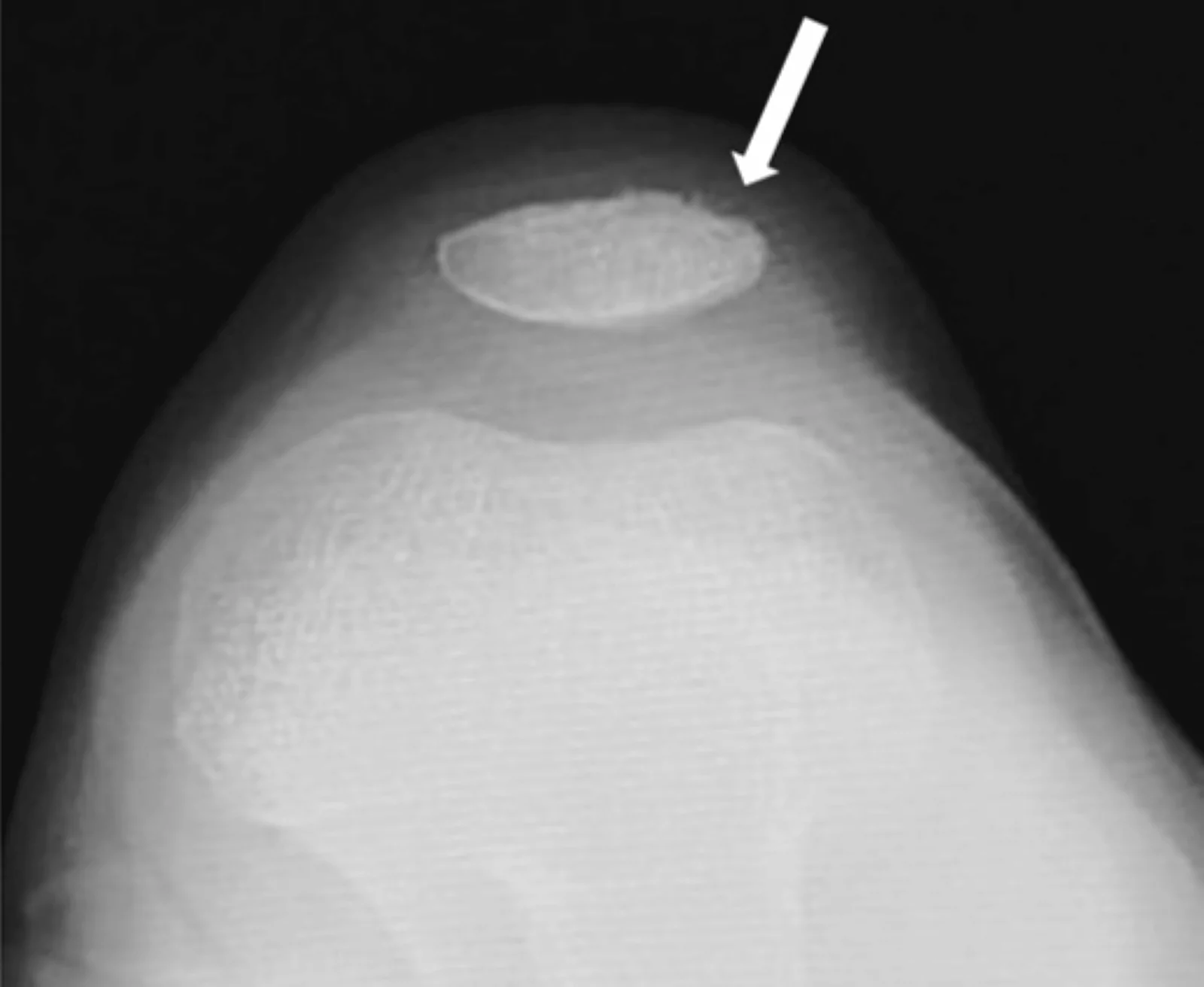
Follow-up radiography immediately before completion of treatment demonstrating resolution of the osteolytic lesion (arrow).

## Discussion

In children presenting with knee pain and periarticular swelling, the differential diagnosis includes traumatic injuries, cellulitis, septic arthritis, osteomyelitis, prepatellar bursitis [3], systemic inflammatory diseases such as juvenile idiopathic arthritis, and neoplastic diseases [6]. Osteomyelitis rarely occurs in sesamoid bones such as the patella. Because of its unique anatomical characteristics and rich vascular supply, bacterial seeding and stagnation within the patella are uncommon [7]. Patellar vascularity develops between 4 and 12 years of age, and trauma during this period of relative vascular immaturity may contribute to the development of patellar osteomyelitis. Patellar osteomyelitis is an uncommon site of infection, representing less than 1% of cases of acute osteomyelitis in children [2]; thus, diagnosis is often difficult and delayed. Children with patellar osteomyelitis frequently require operative procedures, such as drainage, curettage, or sequestrectomy [8].

In this patient, septic arthritis and prepatellar bursitis were excluded on the basis of the clinical and ultrasonographic findings. A provisional diagnosis of prepatellar cellulitis was therefore made, and antimicrobial therapy was initiated. The subsequent growth of MSSA in blood cultures prompted MRI to rule out osteomyelitis of the neighboring long bones, which ultimately led to the identification of concurrent patellar osteomyelitis. Patellar osteomyelitis may develop secondary to contiguous spread of infection from septic bursitis or cellulitis [9]; therefore, presumptive diagnosis of cellulitis alone may result in overlooking concomitant patellar osteomyelitis.

Blood cultures are positive in approximately 40% of cases of pediatric acute osteomyelitis [2], but the positivity rate in patellar osteomyelitis remains unclear because so few cases have been reported. In a previous report of uncomplicated pediatric cellulitis in immunocompetent children, no cases of bacteremia were identified, and obtaining blood cultures on admission was not recommended because it was associated with an approximately 1-day increase in the mean length of hospital stay [10]. However, because some cases of patellar osteomyelitis occur secondary to cellulitis, it is crucial to distinguish isolated cellulitis from patellar osteomyelitis complicated by cellulitis. In the present case, positive blood cultures for MSSA prompted consideration of osteomyelitis involving the adjacent long bones and incidentally led to the diagnosis of patellar osteomyelitis.

If blood cultures had been negative or had not been obtained, MRI evaluation and diagnosis of patellar osteomyelitis might have been delayed, potentially resulting in the need for surgical intervention. MRI, although expensive and occasionally requiring sedation, is the most useful imaging modality for detecting osteomyelitis [2]. However, because patellar osteomyelitis is rare, it may not be immediately considered, and when clinical signs primarily suggest soft tissue infection, as in this case, MRI may be postponed. 

According to the PIDS/IDSA guidelines [5], a three- to four-week antibiotic course is suggested for uncomplicated pediatric acute hematogenous osteomyelitis; however, a longer duration (up to six weeks) is recommended for complicated cases or severe presentations. In our patient, although conservative treatment was successful, the initial presence of MSSA bacteremia and an osteolytic lesion in the left patella prompted us to extend therapy to 6 weeks to ensure complete eradication and minimize the risk of recurrence.

In our patient, conservative treatment with targeted antimicrobial therapy was successfully maintained despite the presence of cortical disruption and cancellous bone defect in the patella. The decision to forgo surgical intervention was supported by several critical clinical criteria: first, the absence of a drainable subperiosteal or intraosseous abscess, as well as the lack of sequestrum formation on serial ultrasonographic and MRI evaluations; second, prompt and favorable clinical response following the initiation of CEZ, evidenced by rapid defervescence and resolution of localized pain; third, steady decline and subsequent normalization of systemic inflammatory markers (CRP and WBC); and finally, confirmed clearance of MSSA bacteremia without persistent positive blood cultures. These findings demonstrate that in selected pediatric patients with acute patellar osteomyelitis, non-operative management can be safely achieved provided there is close monitoring and rapid clinical improvement, even when focal bone destruction is radiologically apparent.

Regarding the pathogenesis, determining the exact route of infection remains unclear due to the simultaneous presentation of prepatellar cellulitis and MSSA bacteremia. The infection may have arisen via contiguous spread from the overlying prepatellar soft tissue inflammation, or conversely, through primary hematogenous seeding into the patellar ossification center with secondary soft tissue involvement. Although distinguishing between these two mechanisms is clinically challenging, the prominent involvement of the patellar ossification center on MRI, coupled with systemic MSSA bacteremia, strongly supports a primary hematogenous origin in line with the general disease concept of pediatric acute hematogenous osteomyelitis [5]. Nevertheless, the possibility of contiguous extension from superficial soft tissue could not be entirely excluded.

This case highlights that patellar osteomyelitis should be considered in the differential diagnosis in children presenting with anterior knee pain, particularly when restriction of patellar mobility is present, and that timely MRI evaluation facilitated prompt diagnosis and helped avoid invasive surgical intervention.

## Conclusions

In children presenting with unilateral knee pain, patellar osteomyelitis should be considered in the differential diagnosis, and timely MRI may facilitate earlier diagnosis and treatment and potentially reduce the need for surgical intervention. Although the blood culture positivity rate in patellar osteomyelitis remains unclear, positive blood cultures may support the diagnosis and help identify the causative organism. Furthermore, as supported by current guidelines for pediatric osteomyelitis, early initiation of appropriate antimicrobial therapy may allow successful resolution without the need for invasive surgical intervention in appropriately selected cases.
